# Outpatient and Home-Based Treatment: Effective Settings for Hip Fracture Rehabilitation in Elderly Patients

**DOI:** 10.3390/geriatrics6030083

**Published:** 2021-08-27

**Authors:** Margarida Mota Freitas, Sara Antunes, Diana Ascenso, Alda Silveira

**Affiliations:** Physical and Rehabilitation Medicine Department, Hospital Garcia de Orta, 2805-267 Almada, Portugal; sara.antunes@hgo.min-saude.pt (S.A.); diana.ascenso@hgo.min-saude.pt (D.A.); maria.silveira@hgo.min-saude.pt (A.S.)

**Keywords:** hip fractures, rehabilitation, aging, frail elderly, activities of daily living

## Abstract

Femoral neck fractures are a major source of disability in the elderly. Rehabilitation is fundamental to recover pre-fracture functionality. We conducted an observational cohort study with the aim of comparing the efficacy of rehabilitation programs in different therapeutic settings. We included elderly patients who had undergone surgical stabilization of a hip fracture. The participants were divided into 3 groups: group 1, outpatient rehabilitation; group 2, inpatient rehabilitation; group 3, home-based rehabilitation. Patients were evaluated at baseline, at three months, and at six months after fracture. Our outcome measures were the Barthel Index (BI), Functional Ambulation Categories, passive and active range of motion of hip flexion and abduction, and muscle strength in hip flexion, abduction, and knee extension. At six months, all three groups showed an average statistically significant improvement (*p* < 0.05) in all outcome measures compared to the baseline. Considering the between-group analysis, final BI was significantly higher in outpatient than inpatient-treated patients (*p* = 0.018), but no statistical difference was found between outpatient and home-based patients. Our findings suggest that rehabilitation leads to significant functional recovery after hip fracture in elderly patients. Both outpatient and home-based rehabilitation seem to be reasonable options for hip fracture rehabilitation.

## 1. Introduction

Hip fractures are the most common osteoporotic fractures, affecting about 18% of women and 6% of men worldwide [1], and are a major concern for the healthcare system and society. Additionally, they might lead to functional impairment and loss of independence in activities of daily living (ADL) [2,3,4,5,6]. Fragility fractures are a serious obstacle to healthy aging, compromising quality of life in affected patients. In Europe, osteoporotic fractures are the fourth leading cause of morbidity associated with chronic disease, contributing annually to over 2.6 million disability-adjusted life years (DALYs), which is more than hypertensive heart disease and rheumatoid arthritis. It is possible to stratify the risk of fracture in osteoporotic patients, and fragility fractures are preventable with pharmacological treatment [7]. Currently, we still lack proper osteoporosis screening programs, particularly for males [8].

Rehabilitation might play a key role on the functional recovery of hip fracture patients, particularly if combined with nutritional supplementation (i.e., vitamin D, calcium, or amino acids) [9,10,11,12,13]. The aims of postsurgical rehabilitation management of hip fractures are to reduce pain, to regain an adequate level of functional activity and social participation, and to improve quality of life. One of the specific acute aims of rehabilitation is to prevent cardiovascular and pulmonary complications. Therefore, lower limb pumping exercises and deep breathing exercises are usually proposed; both activities should be continued until the patient starts walking [14]. Early mobilization (within 24 h post-surgery) might avoid prolonged bed rest and prevent complications such as deep vein thrombosis. It is recommended to maintain muscular strength and endurance of the upper extremities and of the non-operated lower limb. Also, muscle atrophy prevention is important before regaining active mobility and voluntary control of the operated limb [15].

However, there is a big heterogeneity regarding the frequency and timing of the rehabilitation treatment, as well as the choice of the most appropriate setting [16].

Timing of discharge is crucial, considering that the sooner the patient is re-integrated into her/his own environment, the better the functional outcome tends to be [17].

It is mandatory to define the adequate discharge destination for post-hip-fracture patients considering they might undergo rehabilitation in different settings: at home, inpatient, or outpatient facilities [18,19]. The design of dedicated plans is recommended to provide the adequate level of rehabilitation care, and also to prevent recurrent falls and fractures [20].

Inpatient rehabilitation might be a reasonable solution after femoral neck fracture surgical treatment. Patients with heavier comorbidities tend to be selected for inpatient treatment, predicting lower rehabilitation outcomes [21,22].

On the other hand, outpatient facilities might be considered valid settings with attractive functional results in post-hip-fracture patients [23,24]. Outpatient treatment is the most widely available option. It promotes independence and is usually chosen for independent patients.

Lastly, individualized home-based rehabilitation programs might be effective in selected cases. They provide adapted and tailored treatment and can last longer than standard programs, with good functional outcomes [25,26]. The cost of care following a total hip replacement can be significantly reduced by using home-based rehabilitation programs, without compromising the quality of care [27].

To date, there is still a lack of updated information on the role of rehabilitation for femoral neck fracture, considering the setting where it is provided. Therefore, we sought to evaluate the long-term effects of rehabilitation in different settings on disability status and functional outcomes in a pilot sample of post-hip-fracture elderly patients.

## 2. Materials and Methods

### 2.1. Participants

In this real-practice pilot study, we recruited hip fracture patients consecutively admitted to a Traumatology Unit of a Portuguese Hospital in a 3-month period.

Inclusion criteria: (a) age 65 years old and older; (b) patients had undergone surgical stabilization of femoral neck fracture; (c) able to understand and sign the informed consent.

Exclusion criteria: (a) patients not available to attend follow-up appointments for geographic or social reasons; (b) patients with pathological fractures; (c) patients who had comorbidities with an impact on motor, cognitive, or sensory function; (d) patients with cognitive impairment, assessed by a Mini Mental Status Examination (MMSE) < 24/30.

The Hospital Garcia de Orta Ethical Committee approved this study (approval number and date: 37/2020, 1 June 2020). All the participants were asked to carefully read and sign an informed consent. Researchers ensured the confidentiality of study participants and the data collected from them. The study was conducted according to the criteria set by the Declaration of Helsinki, with pertinent National and International regulatory requirements.

### 2.2. Intervention

This is an observational cohort study. The participants were divided into three groups according to their destination after hospital discharge: Group 1 included patients that had been discharged to outpatient rehabilitation treatment; Group 2 included patients referred to inpatient rehabilitation facilities; Group 3 included patients engaged in home-based rehabilitation. Patients were allocated to a certain group according to the decision of the clinical team and not for research purposes. This is an observational study, without interference in the decision of the destination after discharge.

Thus, the rehabilitation therapeutic setting was decided based on several meetings with the patient, family, hospital social worker, nursing, and medical team comprised of an orthopedic surgeon and physiatrist. This team is responsible for discharge planning and has objective clinical and social criteria, such as the will of the patient and her/his family, housing conditions, architectural barriers, previous comorbidities, and functionality and rehabilitation potential.

All patients were examined during a medical appointment two weeks after discharge, at three months and at six months post-fracture. They started physical therapy as soon as possible. Inpatient and home-based programs started in less than two weeks. There was a waiting list for patients attending outpatient rehabilitation and they started physical therapy around one month after discharge. Nevertheless, all patients started physiotherapy sessions about 24 h after surgery, during hospitalization. Also, two weeks after surgery, all patients had a multidisciplinary appointment with a traumatologist and a physiatrist. Patients received information about the exercises they should perform at home to avoid complications and minimize any delay in the rehabilitation process.

To ensure the homogenization of the provided treatment, there was an initial meeting with the physiotherapists. This meeting was attended by physiotherapists who worked in 3 different locations: the outpatient Physical Medicine and Rehabilitation department, inpatient facilities, and a home-based care team.

All patients underwent a similar rehabilitation protocol, consisting of multiple fitness components (aerobic, flexibility, resistance, and neuromotor) based on clinical guidelines for individuals with chronic conditions or functional limitations [28]. In all of the different settings, the exercises were supervised by a physical therapist. In the case of impatient facilities, all these units employed a physiotherapist who could apply the therapeutic program. In the case of home-based patients, a physical therapist from the team was assigned to personally visit patients and apply the same therapeutic program.

The rehabilitation protocol consisted of a set of exercises (walking training, lower limb muscle strengthening, balance exercises, and assisted ambulation) administered three times per week in the first two months, then twice a week for another two months. Most patients were considered healed after four months, based on radiographic signs of bone consolidation, assessed by radiography.

The physical therapy program was divided into four phases:

- Phase 1 (1–2 weeks):
  - Active and active-assisted exercises for hip mobilization;
  - Hip extension exercises, ankle pumps;
  - Active assisted mobilization of the knee, tibiotarsal joint, and contralateral limb;
  - Education about correct positioning at home;
  - Stretching in the Thomas position.
- Phase 2 (3–6 weeks):
  - Maintain previous exercises;
  - Global amplitude gain techniques;
  - Dynamic strengthening;
  - Stretch of the sural triceps, quadriceps, hamstrings, hip flexors
  - Start progressive gait training. Crutches were recommended up to 6 weeks.
- Phase 3 (7–12 weeks):
  - Continue previous exercises;
  - Improve hip range of motion (Objectives: flexion 90°, abduction 30°, extension 0–10°);
  - Concentric-eccentric control;
  - Improve cardiovascular performance;
  - Improve functionality;
  - Gait training (progressively without crutches).
- Phase 4 (3–4 months):
  - Some patients resumed driving;
  - Achieving functional range of motion and good quadriceps control;
  - Increase intensity of previous exercises;
  - Resistance training with static bicycle.

### 2.3. Outcome Measures

At baseline, we collected demographic and clinical data, including MMSE [29], comorbidities, history of previous fragility fractures (hip, vertebral or non-hip non vertebral-NHNV), and the use of anti-osteoporotic drugs or supplementation with vitamin D or calcium.

All patients were examined by a medical doctor of the Rehabilitation team at baseline (T0), at three months (T1), and at six months after fracture (T2). During those medical appointments, we assessed the following outcome measures, all validated for this population:

- Barthel Index (BI), to evaluate the functional independence in ADL (toileting, bathing, eating, dressing, continence, transfers, and ambulation). Each task received a numerical score based on whether the patient required physical assistance to perform it. A patient scoring 0 points would be dependent in all assessed activities of daily living, whereas a score of 100 would reflect independence in all activities [30].
- Functional Ambulation Categories (FAC), to evaluate the independence in ambulation according to six categories ranging from 0 (non-functional ambulation) to 5 (independent walking). Albeit FAC is a general ambulation test, its scores showed a positive linear relationship with gait velocity and step length [31].
- Passive and active range of motion (pROM and aROM) of hip flexion and abduction. Passive range of motion is the movement applied to a joint (by the medical doctor in this case). Active range of motion is movement of a joint provided entirely by the individual performing the exercise, without an outside force aiding in the movement. A goniometer was used to measure all ROMs.
- Medical Research Council (MRC) scale, to evaluate muscle strength in hip flexion, hip abduction, and knee extension [32].

### 2.4. Statistical Analysis

Statistical analysis was performed using STATA v.12 (StataCorp LP, College Station, TX, USA). A study power of 90% was assumed and the statistical significance was defined at 0.05 (α = 0.05). At baseline, the Kruskal Wallis test and the ANOVA test were performed to assess the differences among groups for continuous and categorical variables, respectively. Considering the differences in outcome measures, a between-group analysis was performed using the Wilcoxon rank sum test to compare continuous variables between two groups; Wilcoxon matched-pairs signed rank test for intra-group analysis in all three groups.

## 3. Results

From a total of 52 patients assessed for eligibility, seven patients did not start rehabilitation treatment, eight patients dropped out of the study, and three patients died during the follow-up. Therefore, the final sample consisted of 34 patients (12 male and 40 female), with a mean age of 83.12 ± 7.78 years.

**Figure 1:**
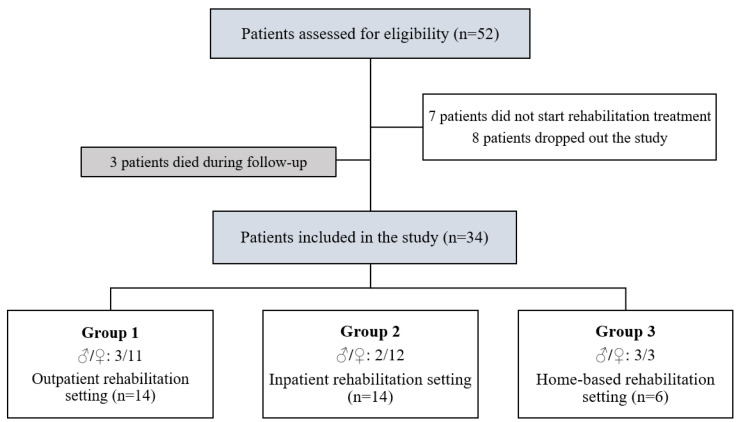
Study flow chart.

The sample was divided into three groups, according to the rehabilitation setting after discharge: 14 patients were referred to outpatient rehabilitation facilities (Group 1), 14 patients were discharged to inpatient facilities (Group 2), and six patients followed integrated home-based rehabilitation care (Group 3) (see the study flow-chart in Figure 1 for further details).

At the baseline (T0), there were no significant differences among groups regarding age, MMSE, comorbidities, previous osteoporotic fractures, osteoporotic treatments (see Table 1 for further details), or functional outcome measures.

All three groups showed a statistically significant improvement (*p* < 0.05) in outcome measures at T1 and T2 compared to baseline, except for MRC of knee extension in Group 3 at 3 months (T0–T1).

There were three statistically significant differences: the mean Barthel index at six months was higher in the outpatient than the inpatient group (88.00 ± 9.73 vs. 68.57 ± 21.70; *p* = 0.018).; Passive hip flexion range of motion at three months was better in the inpatient group than home-based patients (100.36 ± 6.64 vs. 92.50 ± 6.89; *p* = 0.016); hip abduction muscle strength at six months was better in the outpatient group compared to the inpatient group (4.71 ± 0.47 vs. 4.00± 0.96; *p* = 0.030).

The outpatient group showed better improvement in all outcomes at both three and six months, although the significance level was not reached; on the other hand, the inpatient group showed less increase in performance at T1 and T2.

Concerning ROM and muscle strength, Group 1 had better hip aROM and pROM, in both flexion and abduction, and better muscle strength in hip flexors, hip abductors, and knee extensor muscles across all time points (see Table 2 for further details).

Globally, patients treated in outpatient settings showed better functional outcomes, and patients who underwent rehabilitation in inpatient facilities seemed to have worse results. Patients treated at home showed intermediate final functionality, closer to outpatient results.

## 4. Discussion

Community-dwelling hip fracture subjects treated in outpatient rehabilitation settings are more likely to obtain better long-term effects from a rehabilitation protocol regarding functional status, hip ROM, and lower limb muscle strength. Patients referred to home-based rehabilitation showed intermediate results, closer to outpatient settings, even though they were the fastest to achieve rehabilitation treatment after hospital discharge.

All patients started passive mobilization and gait training as soon as possible during hospitalization. Strict cooperation between orthopedic surgeons and physiatrists is needed to provide the best medical care to hip fracture patients worldwide [33,34].

Indeed, a correct evaluation of these patients after surgery might contribute to the decision of an adequate rehabilitation setting. According to our findings, outpatient rehabilitation should be encouraged instead of inpatient rehabilitation settings. Cognitive impairment and confusional states must be excluded, as both have a negative impact on functional outcomes and safety [35]. Our study showed that home-based patients started rehabilitation treatment earlier and had a faster return to their environment, contributing to a better time–space orientation and greater collaboration.

Kauppila’s findings favor home-based programs versus inpatient rehabilitation, following primary total hip replacement [36]. In the same research, validated outcome measures showed no differences in clinical outcomes at 3 and 12 months after surgery.

Our results are in line with the published evidence, even though there were no statistically significant differences between the inpatient and home-based groups. Home-based rehabilitation programs may be the best option in selected social contexts (good family support, possibility of permanent monitoring, motivation).

Considering other studies, we highlight a review by Avola et al. which also investigates the therapeutic effects of rehabilitation programs in femoral neck fractures [37]. However, the papers included in this review differ from ours because they focus on sarcopenia treatment rather than comparing different rehabilitation settings. Some of these studies have similarities with our research because they include the same outcomes, such as the Barthel Index and FAC.

Avola et al. concludes that hip fracture treatment should include specific repetitive exercises and progressive resistance. We adopted this approach to design our study’s rehabilitation program. However, that same review describes the potential advantage of antigravity treadmills, occupational therapy, protein-rich dietary supplementation, erythropoietin, bisphosphonates, calcium, and vitamin D [37]. Knowing the importance of bisphosphonates, calcium, and vitamin D supplementation, we described those as baseline characteristics of our sample. It would have been interesting to our study to collect data on pharmacological treatment and nutritional adaptations that occurred after the fracture.

The strengths of our study include the six-month follow-up, which corresponds to the entire early-rehabilitation period after hip fracture. Also, we had a low dropout rate. As far as we know, this is the first study conducted in Portugal focusing on real-practice characterization of the rehabilitation treatment carried out after hospital discharge in elderly patients after hip fracture. The population was representative of the current clinical practice; there was no manipulation of patient’s treatment assignment, and we used validated scales. We consider that the study protocol did not significantly affect the results.

We are aware that the present pilot study has some limitations. The number of patients was limited by the dimension of the hospital and the Traumatology ward. This small sample size consists of the preliminary results and this study was designed as a real-practice pilot research that aims to give a perspective of Portuguese rehabilitation settings.

While the dimension of the hospital ward limits the number of patients that can be treated in a certain period (and our sample size), it also reflects a common real-life clinical practice in many countries. Due to the need of effective decision-making and resource allocation, our research becomes especially relevant.

The COVID-19 pandemic brought even more pressure to resource management inside and outside the hospital. For many orthopedic services, the local contingency plan meant a reduction on the number of beds and lengths of stay and cancellation of elective surgery [38]. Ultimately, these measures led to early clinical discharges. The pandemic has also had an impact on rehabilitation treatment. There was a reduction in the number of treated patients in both inpatient and outpatient facilities due to COVID-19 infection prevention strategies. Tele-rehabilitation and home-based rehabilitation programs have been developed to ensure continuity of care, ensuring the safety of patients and therapists [38]. Considering this pandemic reality, it is essential to study the therapeutic effectiveness of hip fracture rehabilitation programs in different modalities and settings. Our findings might help the discharge planning team to determine the adequate destination for older people undergoing hip fracture rehabilitation after surgical stabilization.

## 5. Conclusions

Considered together, our findings showed that rehabilitation leads to a significant improvement in functional recovery independently from the setting.

Overall, patients treated on an outpatient basis showed better functional results at six months after hip fracture, compared to patients who received inpatient treatment. Compared to outpatient treatments, patients treated in home-based programs showed slightly lower values on the functionality scales, but without a statistically significant difference. This result points to the hypothesis that both outpatient treatment and home-based treatment are viable options, with positive results.

This real-practice pilot study reported only a cohort of Portuguese rehabilitation care and could be considered as a starting point to further prospective and multi-centric studies on this topic.

Patients’ destination after hospital discharge should be adequately tailored. Rehabilitation professionals should actively participate in the process of discharge planning.

## Figures and Tables

**Table 1:** Sample baseline characteristics.

	Total (*n* = 52)	Outpatient (*n* = 14)	Inpatient (*n* = 14)	Home-Based (*n* = 6)	*p* Value
**Age (years)**	83.12 ± 7.78	81.00 ± 2.50	84.21 ± 2.20	81.17 ± 3.90	0.490
Sex (male/female)	12/40	3/11	2/12	3/3	0.219
**MMSE**	27.41 ± 2.06	27.86 ± 1.83	26.93 ± 2.13	27.5 ± 2.51	0.502
Hypertension	19 (36.53%)	8 (57.14%)	8 (57.14%)	3 (50.00%)	0.950
**Previous myocardial infarction**	7 (13.46%)	3 (21.42%)	2 (14.28%)	2 (33.33%)	0.624
COPD	5 (9.61%)	2 (14.28%)	3 (21.42%)	0 (0.00%)	0.280
**Diabetes**	12 (23.07%)	4 (28.57%)	6 (42.85%)	2 (33.33%)	0.727
Previous fragility fractures	9 (17.31%)	3 (21.42%)	3 (21.42%)	1 (16.67%)	0.966
**Previous hip fractures**	3 (5.77%)	1 (7.14%)	1 (7.14%)	0 (0.00%)	0.796
Vertebral fractures	4 (7.69%)	1 (7.14%)	1 (7.14%)	0 (0.00%)	0.796
**NHNV fractures**	3 (5.77%)	1 (7.14%)	1 (7.14%)	1 (16.67%)	0.757
Anti-osteoporotic drugs or vitamin D or calcium supplementation	10 (19.23%)	1 (7.14%)	4 (28.57%)	2 (33.33%)	0.301
**Bisphosphonates**	8 (15.38%)	1 (7.14%)	4 (28.57%)	1 (16.67%)	0.330
Vitamin D	9 (17.31%)	2 (14.29%)	4 (28.57%)	1 (16.67%)	0.624
**Calcium**	7 (13.46%)	1 (7.14%)	3 (21.42%)	1 (16.67%)	0.560

Continuous variables are expressed as means ± standard deviations; ratios are expressed as x/y; categorical variables are expressed as counts (percentages). The Kruskal Wallis test was performed to assess the differences among groups for continuous variables; The ANOVA test was performed to assess the differences among groups for categorical variables. Abbreviations: MMSE = Mini Mental Status Examination; NHNV = non-femoral non-vertebral; COPD = chronic obstructive pulmonary disease.

**Table 2:** Outcome measures assessed in the three groups at the different times.

Outcomes	Groups	T_0_ Baseline	T_1_ 3 Months	T_2_ 6 Months	*p* Value T_0_–T_1_	*p* Value T_0_–T_2_
**Barthel Index**	Outpatient (*n* = 14)	52.14 ± 8.71	82.86 ± 13.26	88.00 ± 9.73 *	0.001	0.001
	Inpatient (*n* = 14)	50.36 ± 9.29	70.30 ± 19.56	68.57 ± 21.70 *	0.002	0.007
	Home-based (*n* = 6)	54.17 ± 7.36	76.67 ± 16.30	82.50 ± 12.94	0.027	0.026
**FAC**	Outpatient (*n* = 14)	0.00 ± 0.00	3.71 ± 0.94	4.07 ± 0.92	0.001	0.001
	Inpatient (*n* = 14)	0.00 ± 0.00	2.86 ± 1.23	3.14 ± 1.41	0.001	0.001
	Home-based (*n* = 6)	0.00 ± 0.00	3.17 ± 1.17	3.33 ± 1.03	0.027	0.026
**Hip flexion**						
pROM (°)						
	Outpatient (*n* = 14)	28.93 ± 14.17	99.29 ± 10.54	107.14 ± 8.48	0.001	0.001
	Inpatient (*n* = 14)	31.79 ± 17.17	100.36 ± 6.64 *	106.43 ± 9.08	0.001	0.001
	Home-based (*n* = 6)	28.33 ± 16.66	92.50 ± 6.89 *	107.50 ± 4.18	0.027	0.027
aROM (°)						
	Outpatient (*n* = 14)	3.57 ± 3.63	93.57 ± 10.64	99.29 ± 12.07	0.001	0.001
	Inpatient (*n* = 14)	5.00 ± 5.55	93.93 ± 7.38	98.93 ± 9.44	0.001	0.001
	Home-based (*n* = 6)	4.17 ± 4.92	90.00 ± 8.94	99.17 ± 6.65	0.028	0.026
**Hip abduction**						
pROM (°)	Outpatient (*n* = 14)	19.29 ± 10.89	35.71 ± 7.81	36.07 ± 8.13	0.003	0.003
	Inpatient (*n* = 14)	13.93 ± 5.94	29.29 ± 11.24	30.00 ± 12.09	0.001	0.001
	Home-based (*n* = 6)	11.67 ± 4.08	35.00 ± 8.94	35.83 ± 9.70	0.027	0.027
aROM (°)	Outpatient (*n* = 14)	3.21 ± 3.72	27.14 ± 8.93	29.64 ± 8.43	0.001	0.001
	Inpatient (*n* = 14)	3.93 ± 4.01	20.00 ± 9.81	23.57 ± 12.47	0.001	0.002
	Home-based (*n* = 6)	4.17 ± 3.76	27.50 ± 11.73	34.17 ± 8.61	0.028	0.026
**MRC scale**						
Hip flexion	Outpatient (*n* = 14)	1.43 ± 0.65	4.14 ± 0.66	4.71 ± 0.47	0.001	0.001
	Inpatient (*n* = 14)	1.86 ± 0.86	3.86 ± 1.03	4.64 ± 0.50	0.001	0.001
	Home-based (*n* = 6)	1.33 ± 0.52	3.67 ± 0.82	4.83 ± 0.41	0.026	0.024
**MRC scale**						
Hip abduction	Outpatient (*n* = 14)	0.86 ± 0.36	3.50 ± 0.85	4.71 ± 0.47 *	0.001	0.001
	Inpatient (*n* = 14)	0.86 ± 0.36	3.14 ± 1.10	4.00± 0.96 *	0.001	0.001
	Home-based (*n* = 6)	0.83 ± 0.41	3.33 ± 0.52	4.33 ± 0.52	0.024	0.024
Knee extension	Outpatient (*n* = 14)	2.64 ± 0.63	4.07 ± 0.73	4.79 ± 0.43	0.001	0.001
	Inpatient (*n* = 14)	3.00 ± 0.68	3.71 ± 0.73	4.21 ± 0.80	0.008	0.004
	Home-based (*n* = 6)	3.33 ± 0.82	3.83 ± 0.75	4.83 ± 0.41	0.180	0.024

Continuous variables are expressed as means ± standard deviations. Wilcoxon matched-pairs signed rank test was performed for intra-group analysis; Wilcoxon rank sum test was performed for between-group analysis. Abbreviations: FAC: Functional Ambulation Category; pROM: passive Range of Motion; aROM: active Range of Motion; MRC: Medical Research Council. ° represents angle degrees. * = *p* < 0.05 in the between-group analysis.

## Data Availability

The data that support the findings of this study are available from the corresponding author, upon reasonable request.
